# Early *de novo* T cell expansion following SARS-CoV-2 infection predicts favourable clinical and virological outcomes

**DOI:** 10.1016/j.ebiom.2025.105795

**Published:** 2025-06-04

**Authors:** Joe Fenn, Aleksandra Koycheva, Rhia Kundu, Mica Tolosa-Wright, Lulu Wang, Janakan Sam Narean, Emily Conibear, Seran Hakki, Jakob Jonnerby, Samuel Baldwin, Kieran Madon, Sean Nevin, Nieves Derqui, Timesh D. Pillay, Anjna Badhan, Eleanor Parker, Carolina Rosadas, Graham Taylor, Jake Dunning, Ajit Lalvani, INSTINCT Study Investigators, Joe Fenn, Kieran Madon, Emily Conibear, Romain Derelle, Sean Nevin, Rhia Kundu, Seran Hakki, Aleksandra Koycheva, Nieves Derqui, Mica Tolosa-Wright, Jakob Jonnerby, Lulu Wang, Samuel Baldwin, Timesh Pillay, Ryan Thwaites, Constanta Luca, Robert Varro, Anjna Badhan, Eleanor Parker, Carolina Rosadas, Myra McClure, Richard Tedder, Graham Taylor, Ajit Lalvani, Janakan Narean, Lucy Mosscrop, Patricia Watber, Jie Zhou, Jack Barnett, Hamish Houston, Anika Singanayagam, Paul Freemont, Neil Ferguson, Maria Zambon, Wendy Barclay, Jake Dunning, Jessica Cutajar, Valerie Quinn, Sarah Hammett, Eimèar McDermott, Kristel Timcang, Jada Samuel, Samuel Bremang, Samuel Evetts, Megan Davies, Chitra Tejpal, Anjeli Ketkar, Giulia Miserocchi, Harriet Catchpole, Simon Dustan, Isaac Day Weber, Federica Marchesin, Alexandra Kondratiuk

**Affiliations:** a NIHR Health Protection Research Unit in Respiratory Infections, Imperial College London, London, UK; b National Heart and Lung Institute, Imperial College London, London, UK; c MRC Centre for Global Infectious Disease Analysis, School of Public Health, Imperial College London, London, UK; d Department of Infectious Disease, Imperial College London, London, UK; e Pandemic Sciences Institute, University of Oxford, Oxford, UK

**Keywords:** SARS-CoV-2, T cell, Correlates of protection, Household contacts

## Abstract

**Background:**

*De novo* T cell expansion to a novel viral infection is assumed to confer protection, but empirical evidence in humans is limited. The SARS-CoV-2 pandemic provided a unique opportunity to investigate *de novo* T cell-mediated protection in antigen-naïve individuals without the confounding effects of preexisting immune memory.

**Methods:**

We leveraged a prospective household contact study to recruit new COVID-19 cases a median of 4 days post-SARS-CoV-2 exposure. We longitudinally enumerated SARS-CoV-2 antigen-specific functional T cell subsets using dual IFN-γ/IL-2 fluorescence-linked immunospot (FLISpot) assays. We then correlated T cell dynamics with detailed clinical and virological outcomes derived from longitudinal measurement of symptom burden and viral load.

**Findings:**

Early expansion (day 0–7) of SARS-CoV-2-specific IFN-γ-secreting T cells correlated with lower peak viral load and symptom burden. Conversely, late T cell expansion (day 7–28) correlated with higher symptom burden. Neither pre-existing cross-reactive T cells nor early antibody induction correlated with virological outcomes.

**Interpretation:**

These findings provide empiric evidence for early antigen-specific T cell expansion being protective against naturally acquired viral infection in humans.

**Funding:**

This work is supported by the 10.13039/100018336NIHR Health Protection Research Unit in Respiratory Infections, 10.13039/501100000761Imperial College London in partnership with the UK Health Security Agency (Grant number: NIHR200927; AL) and the 10.13039/501100000265Medical Research Council (Grant number: MR/X004058/1).

**Research in context**

**Evidence before this study**

A search of PubMed for manuscripts published in English between 2019 and January 2025 using the terms “T cell” AND (“SARS-CoV-2” OR “COVID-19”) AND (“longitudinal study” OR “prospective study” OR “cohort study” OR “time course”) AND (“clinical outcomes” OR “disease severity” OR “immune correlates” OR “antiviral immunity”)) returned 45 results. These and other related manuscripts have shown that T cell responses in unvaccinated individuals correlate with SARS-CoV-2 disease severity, with delayed and poorly coordinated responses associating with severe clinical outcomes. In vaccinated and previously infected individuals, T cell responses have been shown to associate with superior disease outcomes and viral control. Additionally, our group and others have shown that cross-reactive T cells induced by prior endemic human coronavirus infection protect against SARS-CoV-2 infection in highly exposed contacts.

**Added value of this study**

A central principle of immunology is that *de novo* T cell expansion in response to infection by a newly encountered virus provides protection to the host. Whilst detailed observational and mechanistic studies in animal models have convincingly demonstrated this, empiric evidence in humans is very scarce because of challenges associated with obtaining samples early enough in infection from antigen-naive individuals. Our prospective longitudinal study in newly SARS-CoV-2-infected individuals highlights the pivotal importance of the dynamics of *de novo* responses of T cell functional subsets to SARS-CoV-2 infection. We show that IFN-γ-secreting T cell expansion within the first week of infection can help to contain viral load, whilst later expansion associates with higher symptom burden.

**Implications of all the available evidence**

Whilst pre-existing cross-reactive T cells have a role in protection from SARS-CoV-2 infection, the dynamics of the early *de novo* SARS-CoV-2-specific T cell response appear to have a more pronounced impact on viral load control in infected individuals with mild disease. Our findings provide some of the first empiric data in naturally infected humans in support of the paradigm that very early *de novo* expansion of antigen-specific T cells is required to limit *in vivo* respiratory virus replication.

## Introduction

It is widely believed that early T cell responses to viral infections are protective, limiting disease and viral burden. However, evidence for this in humans is scarce in part because of the presence of pre-existing humoural immunity, which is often protective, and confounds attempts to discern the protective effects of early T cell responses.^1^ Moreover, T cell responses in naturally-acquired viral infections are usually measured when patients present to healthcare facilities, at which point T cell expansion has already occurred and the magnitude of T cell response generally correlates positively with disease burden and viral load, as it is driven by antigen load.^2^^,^^3^ To discern a possible protective role for T cells against viral infection in humans, it is therefore necessary to study immunologically-naïve persons encountering a virus for the first time. Additionally, it is necessary to measure T cell responses very early in infection and longitudinally thereafter in order to prospectively correlate the dynamics of the response with clinical and virological outcomes.

The COVID-19 pandemic provided a unique natural experiment to assess the role of early T cell responses in limiting *in vivo* viral load or symptomatic illness in a population lacking protective antibodies. We recruited and longitudinally followed a community cohort of healthy, unvaccinated SARS-CoV-2 antibody-naïve adults very recently exposed to a newly-diagnosed COVID-19 primary case in the same household. We measured three functional subsets of SARS-CoV-2 antigen-specific T cells and IgG and IgM antibodies at enrolment, on average 4 days post-SARS-CoV-2 exposure and 3 days prior to peak symptom burden (pSB), and serially thereafter up to 28 days. We also serially quantified upper respiratory tract SARS-CoV-2 viral load (VL) and daily symptom burden. This enabled us to correlate early and late expansions of *de novo* SARS-CoV-2 antigen-specific T cells with virological and clinical outcomes in the absence of pre-existing adaptive immunity.

We hypothesised that rapid, early (7-days post enrolment) induction of *de novo* SARS-CoV-2 antigen-specific interferon-gamma (IFN-γ)-secreting T cells would be associated with containment of *in vivo* viral replication and symptom burden while later expansion of more functionally differentiated T cells would correlate positively with symptom burden and viral load. In light of our previous findings showing that cross-reactive T cells induced by prior endemic human coronavirus infection protect against SARS-CoV-2 infection,^4^ we further hypothesised that cross-reactive T cells may also contribute to containment of viral load and symptom burden in individuals who acquire infection. Additionally, we assessed whether induction of *de novo* SARS-CoV-2-specific IgG and IgM antibodies associated with symptom burden and viral load *in vivo*.

## Methods

### Study design and participants

**Fig. 1:**
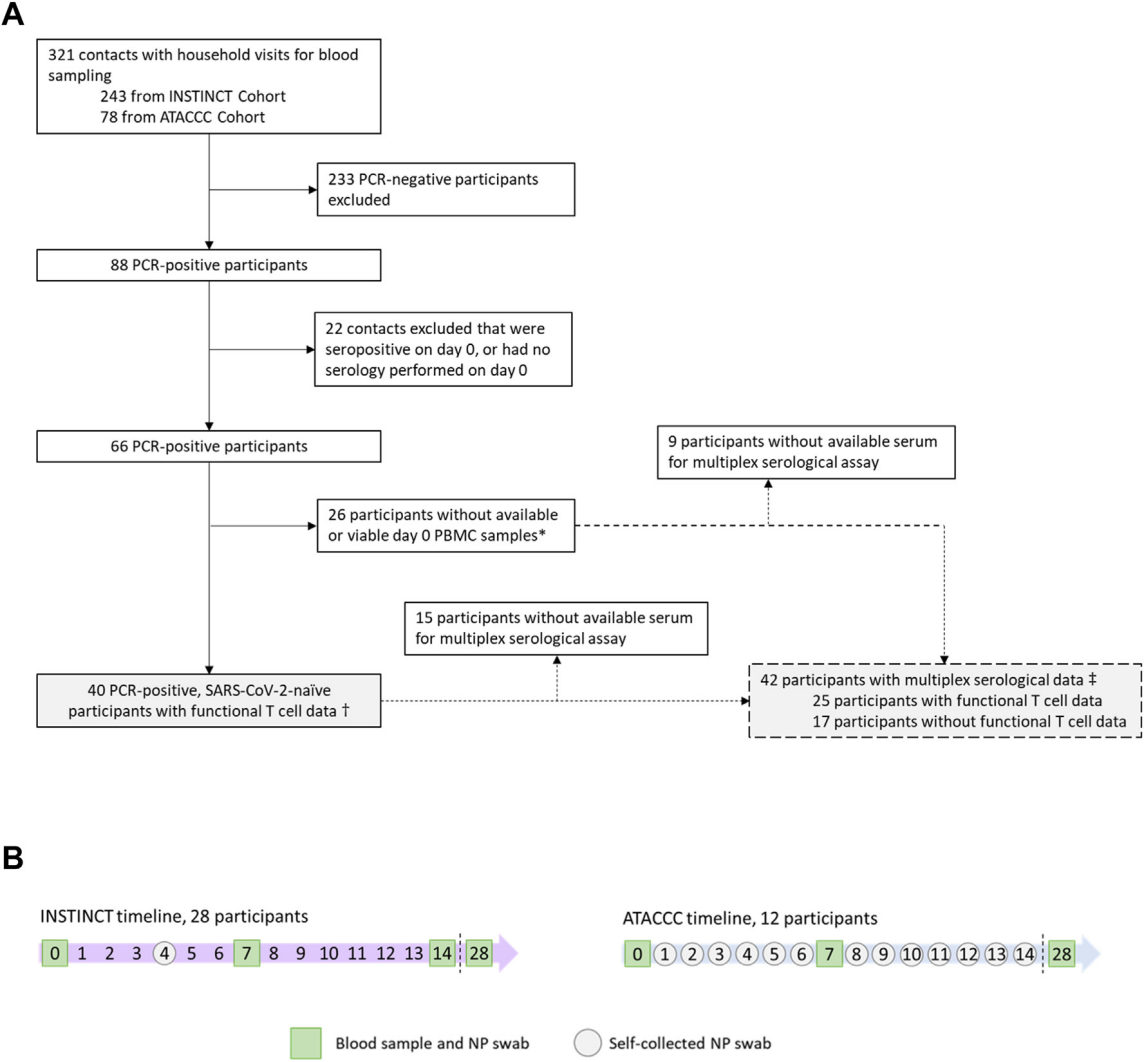
**Inclusion criteria for contacts included in the analysis (A), and INSTINCT and ATACCC recruitment timelines (B)**. INSTINCT, Integrated Network for Surveillance, Trials and Investigations into COVID-19 Transmission. ATACCC, Assessment of Transmission and Contagiousness of COVID-19 in Contacts. All household contacts were enrolled a median of 4 days (IQR 3–5) after first exposure to a symptomatic primary case. Definitions: PCR-positivity was defined as any Ct value < 35 (ATACCC) or VL > 5 copies per PCR reaction (INSTINCT) from a nasopharyngeal (NP) swab in the first 14 days post-enrolment. A sample was considered seropositive if DABA binding ratio ≥1. ∗Non-viable PBMC samples refer to samples that had insufficient viable cells after thawing, or that had no response to the positive controls in the FLISpot assay. †Results shown in Fig. 2, Fig. 3, Fig. 4. ‡Results shown in Fig. 5.

From May 2020 to March 2021, participants were enrolled in two longitudinal community-based observational studies in Greater London, UK: Integrated Network for Surveillance, Trials and Investigations into COVID-19 Transmission (INSTINCT), and Assessment of Transmission And Contagiousness of COVID-19 in Contacts (ATACCC) (Fig. 1A). The studies were approved by the Health Research Authority (20/NW/0231). SARS-CoV-2-infected symptomatic primary cases were identified through the UKHSA national test and trace system and invited to participate alongside their household contacts if the day of enrolment fell within 5 days of primary case symptom onset (median of 4 days [IQR 3–5]). Research nurses visited participants on the day of enrolment (d0), day 7 and 28 to collect blood and nasopharyngeal swabs, and also on day 14 in INSTINCT as previously described.^5^ Additional nasopharyngeal swabs were self-collected by contacts on day 4 (INSTINCT) or on days 1 through 13 (ATACCC) (Fig. 1B). Participants gave written informed consent and were free to leave the study at any time.

At enrolment, participants provided sociodemographic information and listed any symptoms they had experienced in the 5 days preceding enrolment. Following enrolment, daily symptom diaries were collected until d28 which assessed 20 symptoms (Table S2). The presence and severity of each symptom were graded to create a symptom burden (SB) score measuring how unwell participants felt. The highest score was used to generate peak symptom burden (pSB).

### Procedures

Nasopharyngeal swabs were quantified by RT-PCR at Imperial College London (INSTINCT) or UKHSA Colindale (ATACCC), London, UK, with comparable sensitivity and specificity (Supplementary Methods). The threshold for PCR positivity was 6 or more viral copies/RT PCR reaction (equivalent to 240 copies per ml viral transport medium). Serology was performed using a hybrid double antigen binding assay (DABA) for total anti-RBD, as previously described (Supp. Methods). A sample was considered seropositive with a DABA binding ratio result of 1 or higher.

### Dual cytokine fluorescence-linked immunospot (FLISpot) assay

Cryopreserved PBMCs were rested overnight at 37 °C and 5% CO_2_ at high density (1.5 × 10^7^/ml) before stimulating with the cross-reactive peptide pool (Genscript, custom synthetised), comprising 45 peptides (Table S3) identified as cross-reactive between SARS-CoV-2 and either HuCoV-OC43 or HuCoV-HKU1 (Kundu et al., 2022). Cells were also stimulated with 4 protein-spanning peptide pools (15-mers with 11 aa overlap, >70% purity), each representing the spike (S1 and S2), nucleocapsid (N) and membrane (M) proteins (1 μg/peptide/ml) (PM-WCPV-S-2, PM-WCPV-NCAP-2, PM-WCPV-VME-2; JPT, Berlin, Germany). Anti-CD3 and anti-CD28 (100 ng/ml) (FSP-0102-10, Mabtech, Stockholm, Sweden), and CEF (1 μg/ml) (3616-1, Mabtech, Stockholm, Sweden) were used as positive controls, and 0.2% DMSO as the negative control. All timepoints from a participant were included in the same FLISpot run. Stimulated cells were incubated on pre-coated IFN-γ/IL-2 plates (FSP-0102-10, Mabtech, Stockholm, Sweden) for 20 h and processed as per manufacturer's instructions. Spot-forming cells (SFC) were counted using an AID iSpot (AID, Strassberg, Germany) and results were reported as SFCs per million cells after subtraction of the negative control. Day 0 SFCs of a given antigen-specific functional T cell subset were subtracted from day 7 SFCs to calculate ‘early expansion’. Day 7 SFCs were subtracted from day 28 SFCs to calculate ‘late expansion’. IFN-γ- and IL-2-secreting cells refer to IFN-γ-only and IL-2-only SFCs, respectively, excluding dual positive IFN-γ + IL-2 secreting cells. Summated responses for each functional T cell subset are derived from the sum of frequencies of S, M and N pool-specific T cells.

### MesoScale discovery immunoassay

Longitudinal IgG and IgM responses to SARS-CoV-2 Spike, SARS-CoV-2 S1 NTD, SARS-CoV-2 S1 RBD, SARS-CoV-2 N, SARS-CoV-1 Spike, HCoV-HKU1 Spike, HCoV-OC43 Spike, HCoV-NL63 Spike, and HCoV-229E Spike were measured in serum diluted 1:1000, using the MSD V-Plex Coronavirus Panel 2 kit (K15369U, K15370U, Rockville, MD, USA). Samples were quantified on a SQ120 Quickplex instrument and the Discovery Workbench software, as per manufacturer's instructions. Antibody concentrations were calculated using serum-based reference standards and assigned arbitrary units (AU) per mL. Replicates with coefficient of variance higher than 20% were removed from the results.

### Statistical analysis

Statistical analysis was performed using R version 4.3.1 and GraphPad Prism version 10.1.0. Longitudinal comparisons of responses to analytes per functional T cell subset or antibody isotype were performed using mixed-effect analysis with Holm-Sidak correction for multiple testing. The analysis was performed as implemented in GraphPad Prism 10.1.0. This mixed model uses a compound symmetry covariance matrix and is fit using Restricted Maximum Likelihood (REML). In the absence of missing values, this method gives the same P values and multiple comparisons tests as repeated measures ANOVA. Spearman's rank-order correlation was used to investigate associations between T cell frequencies or antibody titres with superior infection outcomes, which were defined as: lower peak viral load (VL), lower VL AUC, lower peak symptom burden score, and higher antibody induction at day 28. Spearman's correlation analysis was preferentially selected over Pearson's correlation analysis because we could not assume that our data was continuous, normally distributed, or that relationships would be linear. Peak viral load values were used only for the participants that peaked post-enrolment and had a peak value over 1000 copies/mL. Viral load AUCs were calculated using the minimum area under the curve trapezoidal rule method.^6^ A p value threshold of less than 0.05 was used for statistical significance.

### Ethics

Ethical approval for this study was obtained from the Northwest - Greater Manchester East Research Ethics Committee in accordance with Health Research Authority regulations (REC Reference: 20/NW/0231 IRAS: 282820). All participants were fully informed about the study's objectives, procedures, potential risks, and benefits and provided written informed consent prior to their inclusion in the study.

### Role of funders

This work is supported by the NIHR Health Protection Research Unit in Respiratory Infections, Imperial College London in partnership with the UK Health Security Agency (Grant number: NIHR200927; AL) and the Medical Research Council (Grant number: MR/X004058/1). Study funders had no role in study design, data collection, data analyses, interpretation, or writing of this report.

## Results

### Characterisation of virological and clinical outcome of recent SARS-CoV-2 infection

**Fig. 2:**
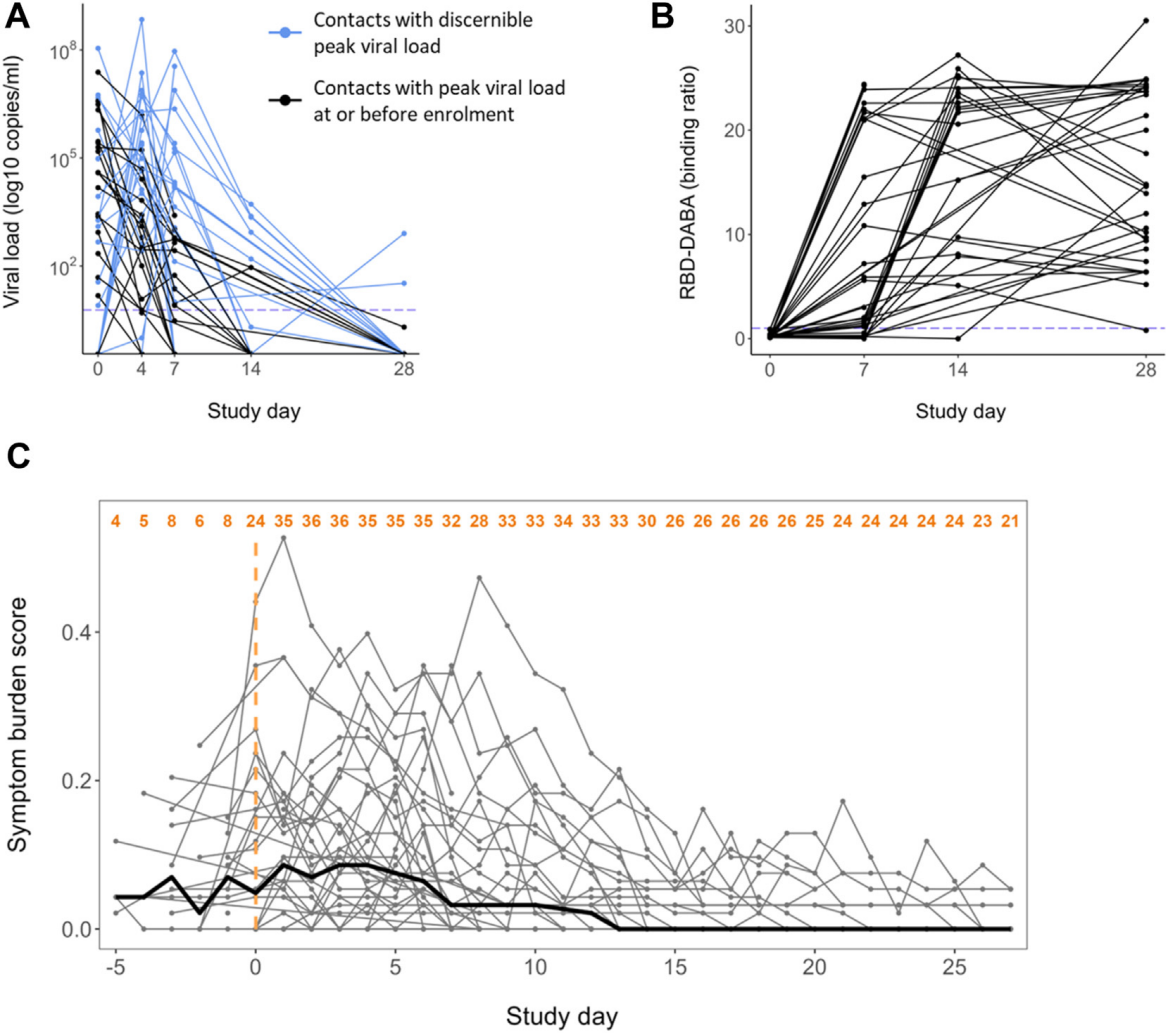
**Characterisation of the study cohort.** Forty recently exposed contacts with no evidence of prior SARS-CoV-2 infection or vaccination were followed longitudinally during the full course of their infection. (A) Viral load trajectories of the cohort. Threshold for positivity is shown with a dotted line at 6 copies/RT-PCR reaction. Blue lines indicate the 19 contacts in whom peak viral load (pVL) could be resolved and used as a virological outcome. Black lines indicate 21 contacts in whom pVL occurred at or before enrolment (d0) and hence pVL could not be reliably determined. (B) Serum IgG and IgM anti-RBD antibodies were measured using a hybrid double antigen binding assay (DABA). Threshold for positivity is shown with a dotted line at binding ratio of 1 arbitrary unit (AU). (C) Individual trajectories of the symptom burden score (in grey) and the median score for the whole cohort each day of observation (in bold black) (n = 38, 2 contacts did not complete comprehensive symptom diaries). The number of participants with symptom scores available for each day is shown in orange at the top. The symptoms comprising the symptom burden score and the scoring are detailed in Table S2.

Our study population comprised 40 unvaccinated, SARS-CoV-2-naïve household contacts of newly-diagnosed PCR-positive primary COVID-19 cases who became infected following exposure (Fig. 1A and B). Demographic characteristics of the cohort are summarised in Table S1. 31 of 40 contacts were SARS-CoV-2 E gene PCR-positive at enrolment (study day 0 (d0)); 9 became PCR-positive post-enrolment (Fig. 2A). All contacts were seronegative at enrolment and seroconverted during the study as determined by total anti-Spike RBD Double Antigen Binding Assay (DABA), confirming their status as previously SARS-CoV-2 antigen-naïve, newly infected individuals (Fig. 2B). 38 of 40 contacts completed comprehensive daily symptom diaries enabling a symptom burden score to be calculated (Table S2). In symptomatic contacts symptom burden peak occurred at a median of 3 days post-enrolment (IQR 1–6) (Fig. 2C). Four contacts were asymptomatic throughout the study.

### SARS-CoV-2-specififc IFN-γ secreting T cells expand rapidly and transiently after infection while IL-2-secreting T cells expand later, remaining elevated at d28

We stimulated PBMCs collected at days 0, 7, 14 and 28 post-enrolment with overlapping 15-mer peptide pools spanning SARS-CoV-2 spike (S), membrane (M), and nucleocapsid (N) proteins, in addition to a pool comprising peptides representing endemic human coronavirus (HuCoV)/SARS-CoV-2 cross-reactive epitopes (Table S3), before performing dual IFN-γ/IL-2 fluorescence-linked immunospot (FLISpot) assays.

**Fig. 3:**
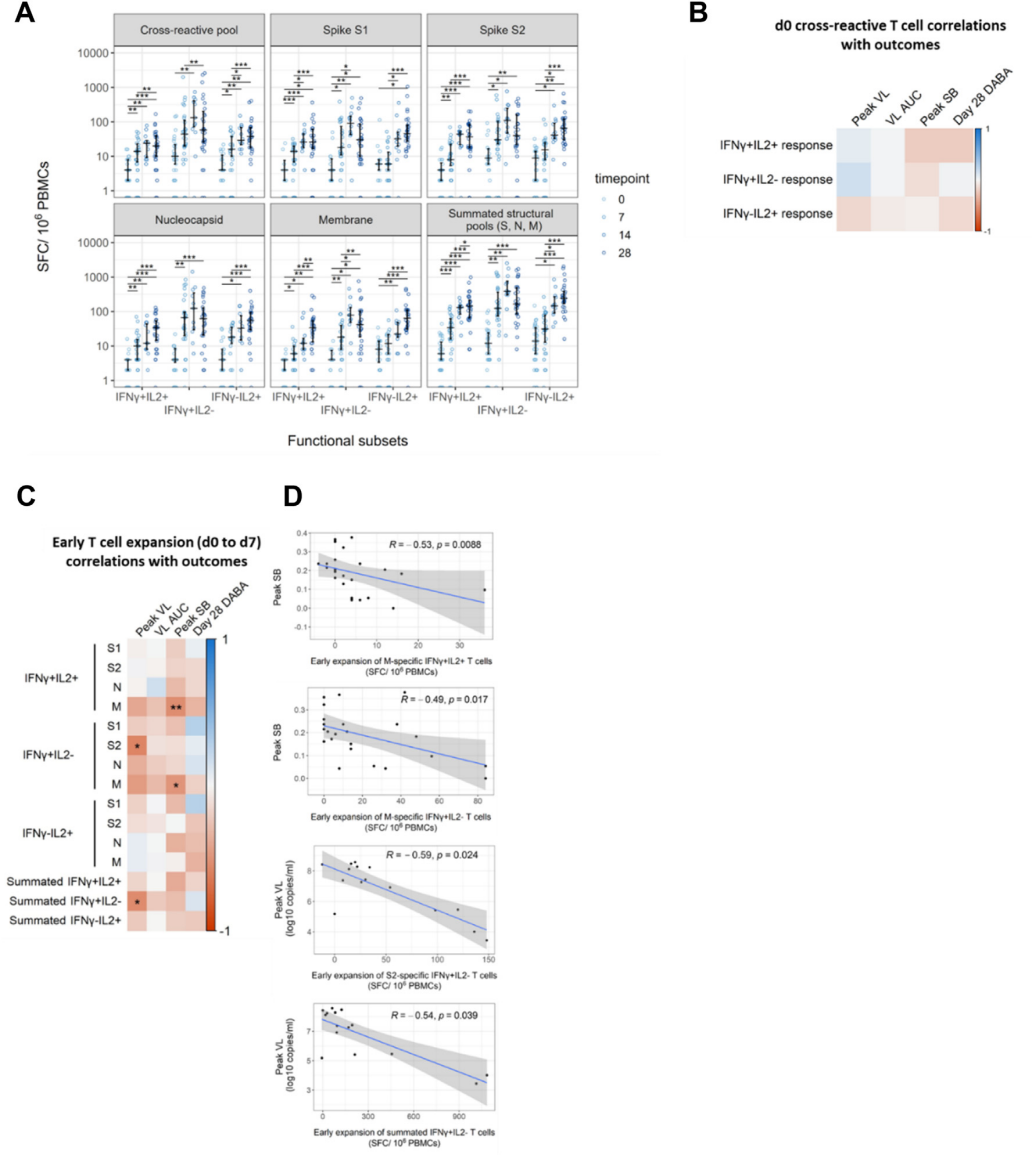
**Early *de novo* induction of SARS-CoV-2 specific T cells in recently exposed, PCR-positive contacts correlate with favourable infection outcomes.** (A) Early *de novo* induction of SARS-CoV-2 specific T cells in recently exposed, PCR-positive contacts correlate with favourable infection outcomes. Longitudinal frequencies of cytokine-producing T cells were measured using a FLISpot assay. Spot-forming cells (SFC) per million PBMCs are displayed stratified by peptide pool and functional subset. Comparisons between timepoints were performed using mixed-effect analysis with Holm-Sidak correction for multiple testing. Summated responses for each functional T cell subset are derived from the sum of frequencies of S, M and N pool-specific T cells. Number of replicates at each time point varies due to random loss to follow-up and limited d14 sample collection d0 n = 40, d7 n = 30, d14 n = 12, d28 n = 29. (B) Heatmap depicting Spearman's analysis demonstrating lack of significant correlations between frequencies of cross-reactive peptide-responsive T cells at d0 and peak VL (n = 21), VL AUC (n = 40), peak SB (n = 38) and DABA at d28 (n = 33). (C) Heatmap depicting Spearman's analysis demonstrating significant correlation between early expansion (d0 to d7) of T cells specific for structural protein-derived peptides with peak VL (n = 16), VL AUC (n = 28), peak SB (n = 26) and DABA at d28 (n = 22). (D) Scatter plots depicting significant associations between early expansion of T cell subsets and peak SB or peak VL identified in Fig. 3C. Line of best fit and 95% confidence interval are displayed.

SARS-CoV-2 antigen-specific T cells expanded significantly following infection (Fig. 3A). S, M and N-specific IFN-γ-only-secreting T cells (hereafter IFN-γ-secreting T cells) and dual IFN-γ and IL-2-secreting T-cells expanded significantly between study days 0 and 7 (d0 and d7). IL-2-only-secreting T cells (hereafter IL-2-secreting T cells) expanded later, with significant increases from d0 frequencies of S, M and N-specific IL-2-secreting cells first evident at d14. We observed a trend towards contraction of IFN-γ-secreting T cell frequency between d14 and d28 whereas frequencies of IL-2-secreting T cells continued to increase from d14 to d28.

### Pre-existing cross-reactive T cells specific for conserved epitopes did not correlate with virological or clinical infection outcomes

Prior evidence from our group and others shows that pre-existing IL-2-secreting T cells from prior endemic human coronavirus infection can cross-recognize SARS-CoV-2 and may protect against infection despite exposure.^1^^,^^4^ We therefore investigated whether these pre-existing cross-reactive T cells also associate with superior control of SARS-CoV-2 viral load or symptom burden in recently infected contacts. Spearman's rank correlation analysis showed that the frequency of cross-reactive IL-2-secreting T cell at d0 did not correlate significantly with pVL or pSB (Fig. 3B). Similarly, frequencies of the other functional subsets of pre-existing cross-reactive T cells did not correlate significantly with viral load or symptom burden (Fig. 3B, Fig. S1).

Only 6 of 40 participants had T cell responses to the cross-reactive pool above the threshold of 22 spot-forming cells (SFC)/10^6^ PBMC described in Kundu et al.^4^ Similarly to the wider cohort, in a subset analysis of these 6 individuals there were no significant correlations between antigen-specific T cell frequencies and outcomes (data not shown).

### Early expansion of SARS-CoV-2-specific IFN-γ secreting T cells correlates with reduced viral load and lower symptom burden

We next investigated the relationship between early *de novo* T cell expansion, defined as d7 frequency of a given antigen-specific functional T cell subset minus the corresponding d0 frequency, and virological and clinical infection outcomes (Fig. 3C and D). Of the 40 enrolled contacts, 30 had paired d0 and d7 FLISpot data available. Of these, the highest measured viral load was at d0 in 14 contacts; accurate resolution of peak viral load (pVL) was therefore only possible in the remaining 16 contacts who were recruited while their viral load was still on an upward trajectory (Fig. 2A).

Using data from these 16 contacts with resolvable pVL, who shared almost synchronous viral load trajectories (Fig. 2A), we tested whether early *de novo* T cell induction predicted a lower peak VL. Consistent with our hypothesis, early T cell expansion correlated negatively with pVL (Fig. 3C). Notably, early expansion of summated IFN-γ-secreting T cells (derived from the sum of S, M and N-specific T cells) correlated negatively and significantly with pVL (Fig. 3C and D). This association was at least partially driven by early S2-specific IFN-γ-secreting T cell expansion which also correlated significantly and negatively with pVL. Additionally, we observed that early expansion of M-specific T cells (both IFN-γ and dual positive IFN-γ and IL-2-secreting cells) correlated inversely with pSB (Fig. 3C and D).

### Early T cell expansion inversely correlates with late T cell expansion

**Fig. 4:**
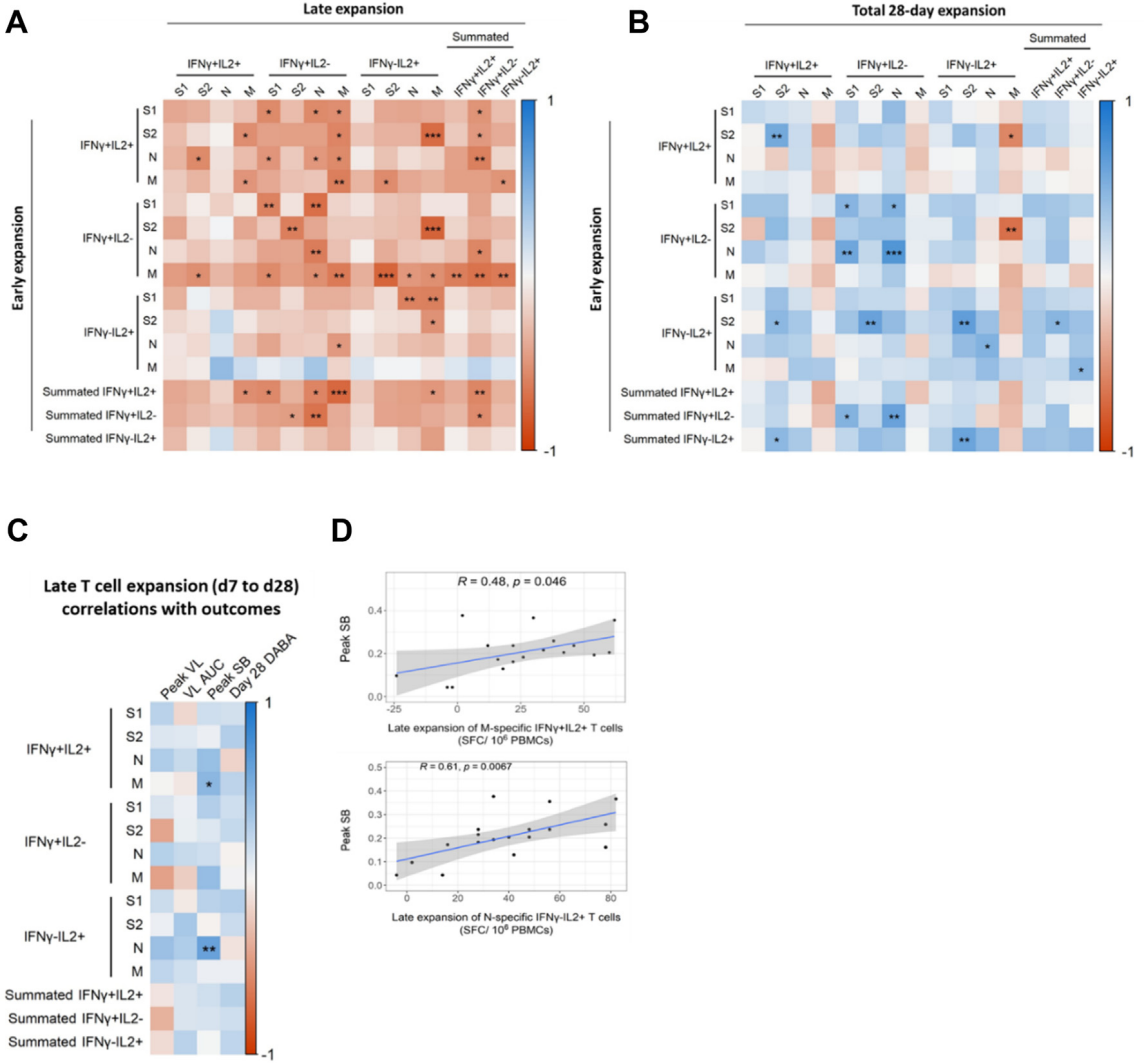
**Early expansion of antigen-specific T cells predicts magnitude of later expansion.** (A) Heatmap depicting Spearman's analysis of correlations between early (d0–d7) and late (d7–d28) T cell expansion. (B) Heatmap depicting Spearman's analysis of correlations between early (d0–d7) and total 28-day (d0–d28) T cell expansion. Summated responses for each functional T cell subset are derived from the sum of frequencies of S, M and N pool-specific T cells. In all plots, statistically significant correlations are indicated by asterisks (∗p ≤ 0.05, ∗∗p ≤ 0.01, ∗∗∗p ≤ 0.001, ∗∗∗∗p ≤ 0.0001). Heatmap colour represents Spearman's R value. Blue indicates a positive correlation whilst red indicates a negative correlation. (C) Heatmap depicting Spearman's analysis demonstrating significant correlation between late expansion (d7 to d28) of T cells specific for structural protein-derived peptides with peak VL (n = 10), VL AUC (n = 20), peak SB (n = 20) and DABA at d28 (n = 20). (D) Scatter plots depicting significant associations between early expansion of T cell subsets and peak SB identified in Fig. 3E. Line of best fit and 95% confidence interval are displayed. In all plots, statistically significant correlations are indicated by asterisks (∗p ≤ 0.05, ∗∗p ≤ 0.01, ∗∗∗p ≤ 0.001, ∗∗∗∗p ≤ 0.0001). Heatmap colour represents Spearman's R value. Blue indicates a positive correlation whilst red indicates a negative correlation.

Early antigen-specific T-cell expansion (between d0 and d7) correlated inversely with late expansion, defined as the d28 frequency of a given antigen-specific functional T cell subset minus the corresponding d7 frequency (Fig. 4A). Early expansion of IFN-γ and dual positive IFN-γ- and IL-2-secreting T cells strongly predicted less late expansion of IFN-γ-secreting T cells. Additionally, early expansion of a given functional subset tended to correlate positively with total 28-day expansion (defined as d28 frequency of a given antigen-specific functional T cell subset minus the corresponding d0 frequency); hence, earlier T cell expansion associated with less late T cell expansion, but higher total 28-day expansion between d0 and d28 (Fig. 4B). Interestingly, however, early expansion of S2-specific IL-2-secreting T cells correlated with higher total 28-day expansion of IFN-γ and dual positive IFN-γ and IL-2-secreting T cells specific to the same antigen.

### Late T cell expansion correlates with increased peak symptom burden

We next tested our hypothesis that late T cell expansion of more functionally differentiated T cells would correlate positively with symptom burden and viral load. Late expansion of M-specific dual positive IFN-γ and IL-2-secreting T cells correlated positively with pSB (Fig. 4C and D), supporting our hypothesis. This is in direct contrast with the negative correlation between *early* expansion of this same M-specific dual IFN-γ/IL-2 positive functional T cell subset and pSB (Fig. 3C). Late N-specific IL-2-secreting T cell expansion also significantly positively correlated with pSB (Fig. 4C and D), as did total 28-day N-specific IL-2 and dual IFN-γ/IL-2 positive secreting T cell frequency (Fig. S2). This is consistent with prior observations of protracted activation and proliferation of T cells in severe compared to mild COVID-19.^7^^,^^8^ Though our observational study design precludes mechanistic conclusions, our findings are consistent with a failure in early T cell expansion permitting higher viral and antigen load. We speculate that this could drive high-magnitude later T cell expansion and associated inflammation which may contribute to increased symptom burden.^9^

### Early *de novo* antibody production does not correlate with superior viral control

Although our central hypothesis was that rapidly induced IFN-γ-secreting T cells would limit viral replication, we also assessed whether rapid antibody induction might also predict viral and clinical outcomes. To test this, we investigated the dynamics of *de novo* antibody induction and their associations with pVL and VL AUC, as well as pSB. Concentration of HuCoV and SARS-CoV-2 antigen-specific IgG and IgM were quantified longitudinally in 42 recently infected household contacts of primary COVID-19 cases using MesoScale Discovery (MSD) V-Plex Coronavirus Panel assay. All 42 contacts were seronegative at enrolment and seroconverted during follow up. Of these, 25 individuals overlap with the cohort of 40 in whom T cell FLISpot was performed (Fig. 1A).

**Fig. 5:**
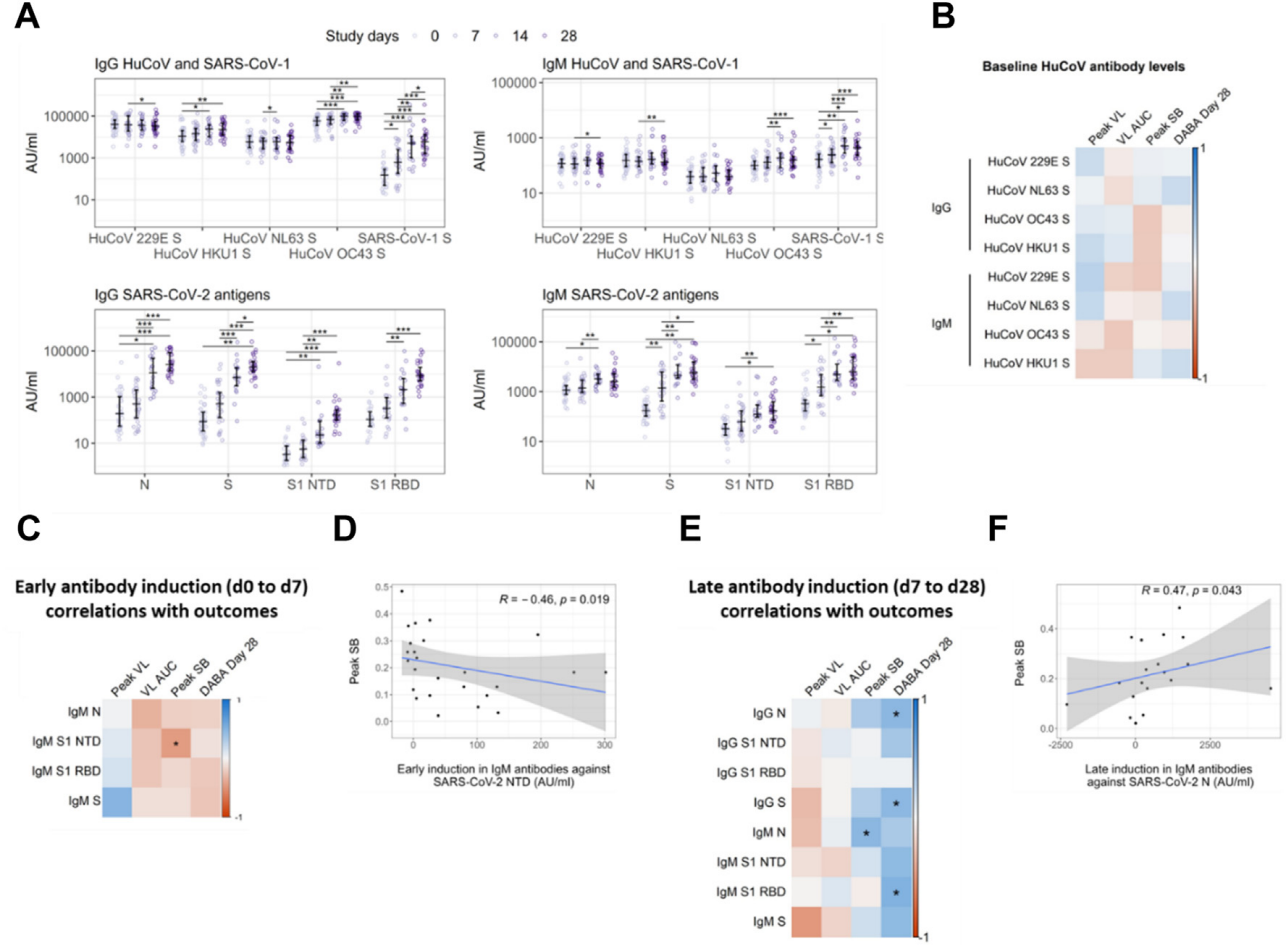
**SARS-CoV-2-specific antibody induction in previously SARS-CoV-2-naïve participants during acute infection.** (A) Longitudinal antibody titres against SARS-CoV-2 and HuCoV antigens, grouped by isotype, were measured using an MSD V-plex assay. Comparisons between timepoints were performed using mixed-effect analysis with Holm-Sidak correction for multiple testing. (B) Spearman's correlation analysis of antibody titres against HuCoVs at d0 with peak VL (n = 22), VL AUC (n = 42), peak SB (n = 41) and DABA at d28 (n = 28). (C) Spearman's correlation analysis of early antibody induction, defined as the induction between d0 and 7, between all IgM titres and peak VL (n = 12), VL AUC (n = 29), peak SB (n = 29) and DABA at d28 (n = 21). A single outlier with early S1 NTD-specific IgM induction >3 standard deviations above the mean (2190 AU) was excluded in these analyses. (D) Correlation between early induction of anti-S1 NTD IgM and peak SB is shown. Spearman's R and p values are displayed (R and p values with inclusion of outlier are 0.51 and 0.0072 respectivly). (E) Spearman's correlation analysis of late antibody induction, defined as the induction from d7 to 28, between all IgM and IgG titres and peak VL (n = 10), VL AUC (n = 22), peak SB (n = 22) and DABA at d28 (n = 21). A single outlier with early S1 NTD-specific IgM induction >3 standard deviations above the mean (35000 AU) was excluded in these analyses. (F) Correlation between late induction of anti-N IgM and peak SB is shown. (R and p values with inclusion of outlier are 0.48 and 0.032 respectivly). In all plots, statistically significant correlations are indicated by asterisks (∗p ≤ 0.05, ∗∗p ≤ 0.01, ∗∗∗p ≤ 0.001, ∗∗∗∗p ≤ 0.0001). Heatmap colour represents Spearman's R value. Blue indicates a positive correlation whilst red indicates a negative correlation.

Anti-HKU1, OC43 and SARS-CoV-1 S antibody titres increased significantly following infection, as previously reported after both natural infection and SARS-CoV-2 vaccination^10^, ^11^, ^12^ (Fig. 5A). This observation is likely to be caused by the induction of cross-reactive antibodies, concordant with the higher sequence homology, and therefore likely increased frequency of shared epitopes, between SARS-CoV-2 S and HKU1, OC43, and SARS-CoV-1 S compared to 229E and NL63 S.^4^^,^^13^ Such potential cross-reactivity likely arises through newly generated SARS-CoV-2 specific antibodies reacting with the conserved epitopes from related viruses. Alternatively, the increase in antibody titres to related viruses may result from reactivation of memory B cells originally generated against seasonal coronaviruses, though this would not explain the increase in antibody titres to SARS-CoV-1 which our cohort is very unlikely to have encountered. Although these data support the notion that HuCoV S-reactive antibodies cross-recognise SARS-CoV-2 antigens, we observed no correlation between d0 anti-HuCoV S antibody titre and infection outcomes including viral load and symptom burden (Fig. 5B). Hence, our data do not support a protective role for pre-existing anti-HuCoV antibodies in individuals with SARS-CoV-2 infection.

Titres of SARS-CoV-2 N and S-specific IgG and IgM increased significantly following infection. N and S specific IgG was undetectable at d7 in most cases with significant increases from baseline IgG titre first observed at d14. IgM titres increased more rapidly, with significant increases from baseline observed by d7 (Fig. 5A). As for antigen-specific T cell expansion, we investigated relationships between antibody dynamics and virological and clinical outcome using multiple Spearman's correlation analysis. As no early induction of SARS-CoV-2-specific IgG was observed, we did not investigate associations between this parameter and outcomes. However, early induction of S1 NTD-specific IgM did correlate significantly and negatively with pSB suggesting a potentially clinically relevant protective effect (Fig. 5C and D). This significant association was conserved whether or not an outlier with early S1 NTD-specific IgM induction >3 standard deviations above the mean was included in the analyses. There were no significant associations between early antibody induction and virological parameters.

In contrast to the potentially protective effect of early IgM induction, late induction of N-specific IgM correlated positively with peak symptom burden (Fig. 5E and F). We also observed a positive correlation between the late induction of anti-N and anti-S IgG and anti-S1 RBD IgM and d28 DABA results, showing concordance between the two methods of quantifying anti-SARS-CoV-2 antibody responses. There were no significant associations between late antibody induction and virological parameters.

## Discussion

Whilst pre-existing cross-reactive T cells have been convincingly shown to protect against SARS-CoV-2 infection,^1^^,^^4^ our data does not support a significant effect of these cells on containing established infection. We did, however observe associations between expansion of *de novo* SARS-CoV-2 specific T cells and superior control of SARS-CoV-2. Specifically, we found that early IFN-γ-secreting T cells expansion predicted lower pVL, supporting our original hypothesis that rapid *de novo* T cell expansion would improve viral containment. Importantly, it constitutes empiric evidence for the speed of induction of antigen-specific T cell responses associating with protection against naturally acquired viral infection in humans which has previously been limited due to challenges related to timing of sample collection and the confounding effects of preexisting adaptive immune memory.

Our findings build upon prior studies that have suggested associations between T cell response and control of SARS-CoV-2 replication. A study of 12 hospitalised COVID-19 patients early in the pandemic showed that an earlier first day of detection of IFNγ-secreting SARS-CoV-2-specific T cells associated with shorter duration of infection, suggesting associations between the timing of T cell induction and viral control across the clinical spectrum of COVID-19 outcomes.^14^ Ramirez et al. recruited a cohort of community COVID-19 cases at around 1 week post symptom onset and showed that magnitude of the SARS-CoV-2-specific CD8+ T cell IFN-γ response at enrolment associated with lower concomitant viral load.^15^ They also showed that frequencies of these IFN-γ-secreting cells remained stable for 28 days from enrolment whereas IL-2-secreting T cell responses increased, meaning that IL-2 responses developed relatively more slowly compared to IFN-γ-secreting T cell expansion which had already occurred by enrolment. Our household contact study design enabled enrolment of cases within days of exposure to an index case and often before peak viral load enabling detailed assessment of the relationship between viral load dynamics and both the timing and magnitude of T cell expansion across the course of infection in mild ambulatory COVID-19 cases. As such, we were able to provide empiric evidence that IFN-γ-secreting T cell responses are formed early during primary infection and associate with viral control whereas development of IL-2 responses occurs relatively later in infection.

Interestingly, our findings are compatible with observations from a controlled human infection model (CHIM) study of SARS-CoV-2 infection.^16^ Wagstaffe et al. showed that peak size of the activated CD8+ T cell population correlated negatively with nasal viral load area under the curve (AUC). This correlation, given that CD8+ T cells are typically the primary producers of IFN-γ during *de novo* T cell responses, aligns with our observations of a protective effect of IFN-γ-secreting T cells.^16^^,^^17^ We do, however, acknowledge that both CD4+ and CD8+ T cells may have contributed to the protective IFN-γ-secreting T cell population we identified and that the difference in the methods used to quantify T cell populations in the two studies limits our ability to directly compare between them.

Studies of vaccine breakthrough SARS-CoV-2 infections further support the notion that early, rapid T cell expansion is associated with improved control of viral load. Spike-specific CD4 and CD8 T cells expand more rapidly in vaccinated individuals with breakthrough Omicron variant infections who more effectively control SARS-CoV-2 viral load compared to unvaccinated individuals.^18^ Furthermore, activated S-specific CD8+ but not CD4+ T cell frequency in the first days of infection correlates negatively with peak viral load and positively with viral clearance rate.^19^ Our study of real-world primary SARS-CoV-2 infection, CHIM studies of primary infection and studies of breakthrough infections in vaccinated individuals all converge on the finding that a subset of expanding IFNγ-secreting or CD8+ T cells are associated with superior viral control. That these varied study designs and distinct but complementary assays, i.e. FLISpot and flow cytometry respectively, reach similar conclusions supports the robustness and generalisability of the observation.

Our observation of rapid IFN-γ-secreting T cell expansion following SARS-CoV-2 infection and contract within 28 days of infection is consistent with the well-characterised rapid acquisition of CD8+ T cell T_H_1 effector function following short-term antigen stimulation in *in vivo* infection models.^17^^,^^20^ In contrast, more highly differentiated immune memory-associated IL-2-secreting T cells exhibited a delayed but sustained expansion, remaining elevated 28 days post-infection, which likely contributes to the formation of a durable pool of CD4+ memory T cells.^20^^,^^21^ These findings are consistent with prior observations of contraction of IFN-γ-secreting T cell responses following acute SARS-CoV-2 infection and persistence of IL-2 secreting potential of SARS-CoV-2-specific T cells months after initial infection.^9^^,^^22^

That early expansion of IL-2-secreting T cells specific to a given antigen correlated positively with later expansion of IFN-γ and dual IFN-γ and IL-2-secreting T cells specific to the same antigen. This is consistent with the anticipated clonal expansion and functional differentiation of IL-2-secreting antigen-specific T cells to more differentiated effector memory cells capable of IFN-γ production.^17^^,^^20^^,^^21^ The inverse relationship between overall early and late T cell expansions, combined with the negative association of early T cell expansion with pVL, suggests that early T cell expansion can limit viral and antigen load, consequently reducing later antigen-driven T cell expansion.

We observed no association between antibody production and improved viral control, though early induction of S1 NTD-specific IgM correlated with superior clinical outcome. The extent to which this protective association extends beyond mild disease is unknown, though there is a consensus that robust early antibody responses are advantageous.^23^ In contrast to the potentially protective effect of early IgM induction, late induction of N-specific IgM correlated positively with peak symptom burden, consistent with prior evidence of positive associations between antibody titre and protracted antigen load typical of more severe COVID-19 disease.^24^^,^^25^

Our study has certain limitations. The diversity of clinical outcomes in our study was limited as a natural consequence of recruiting community COVID-19 cases the vast majority of whom had mild and self-resolving illness. As a result, the extent to which our findings can be generalised to more severe COVID-19 cases is unknown. The extent to which findings can be generalised to older adults and children or those with multiple comorbidities is also unknown. Due to the limited sample size this study is underpowered to definitively state how age, sex, co-morbidity, and other demographic factors affect the observations. We did not perform multivariate or formal subset analyses to investigate potential confounding effects of factors like ethnicity, age and sex. In exploratory analyses of the effect of sex no significant differences between sexes in terms of frequencies of T cells specific to any of the antigens investigated were found at any timepoint. Future similarly designed studies with larger cohorts could be used to generate datasets more amenable to subgroup and multivariate analyses to better understand the potentially confounding effects of demographic factors.

We acknowledge the potential for self-reporting bias as participants' perceptions and reporting of symptoms may have been influenced by subjective factors, potentially affecting some of our inferences. More granular sample collection would enable more accurate definition of pVLs and improved resolution of the dynamics of T cell responses. Finally, it is important to note that our classifications of the phenotype and differentiation status of these T cell subsets are based solely on their functionality and that cell surface marker phenotyping by flow cytometry was not performed on antigen-stimulated T cells in this study due to limited availability of sufficient cell numbers.

Our data support a role for early antigen-specific T cell expansion in control of viral replication *in vivo*, and in reducing symptoms. In contrast, later expansion of T cells, from the second week of infection onwards, correlated positively with symptom burden. This may be because late expansion of antigen-specific T cells is driven by a continued increase in antigen load due to poor initial control of viral replication. Notably, early expansion of a given antigen-specific T cell functional subset (SARS-CoV-2 M-specific IFN-γ and IL-2-secreting T cells) correlated with better clinical outcome while later expansion of the same T cell subset correlated with poorer clinical outcome.

These findings highlight the pivotal importance of the speed of induction of cellular immune responses to viral infection. Our data suggest that expansion within the first week of infection can help to contain viral load and symptom burden, while later expansion is not protective and is associated with poorer outcomes. The widely held assumption that very early *de novo* expansion of antigen-specific T cells is required to limit viral replication *in vivo* has been based on several animal models of diverse viral infections. Our empiric data from the recent pandemic provide important evidence that this fundamental immunological paradigm likely pertains in naturally infected humans.

## Contributors

Conceptualisation: JF, RK, AL.

Software: JF, AK, RK.

Validation: JF, AK, RK, MTW, LW, JSN.

Formal analysis: JF, AK, RK.

Investigation: JF, AK, RK, MTW, LW, JSN, EC, SH, JJ, SB, KM, SN, ND, TDP, AB, EP, CR, GT, JD, AL.

Data curation: JF, AK, RK, MTW, LW, JSN.

Writing—original draft: JF, AK.

Writing—review & editing: JF, AK, RK, EC, SH, JJ, SB, KM, SN, GT, JD, AL.

Visualization: JF, AK.

Supervision: JF, RK, GT, JD, AL.

Project administration: AL.

Funding acquisition: JD, AL.

The following authors accessed and verified that data underlying data presented in this manuscript: JF, AK, AL.

INSTINCT study group investigators contributed to participant recruitment and enrolment, collection and biobanking of biological samples, and management, administrative and logistical aspects of the INSTINCT study.

Authors have read and approved the final version of this manuscript.

## Data sharing statement

Anonymised, de-identified versions of datasets used to produce graphs and tables presented in this manuscript are available with investigator support upon reasonable request to the corresponding author (jfenn@ic.ac.uk) following publication. INSTINCT study documents including study protocol, template participant case record form (CRF), informed consent form and participant information sheet (PIS) are also available upon request.

## Declaration of interests

The authors declare no competing interests. The INSTINCT study group was funded by NIHR as part of the Health Protection Research Unit in Respiratory Infections (NIHR200927).

## Appendix A Supplementary data

Supplemental Material

INSTINCT Study Investigators
