# Supplementary material for: Early de novo T cell expansion following SARS-CoV-2 infection predicts favourable clinical and virological outcomes
Source: eBioMedicine. 2025 Jun 4;117:105795. doi: 10.1016/j.ebiom.2025.105795 (PMC12171543; doi:10.1016/j.ebiom.2025.105795)
Supplement: Supplemental Material [file mmc1.docx]

**Supplementary content**

**Supplementary Methods**

Laboratory assays

Further sociodemographic and clinical data

**Supplementary Tables**

Supplementary Table 1

Supplementary Table 2

Supplementary Table 3

**Supplementary Figures**

Supplementary Figure 1

Supplementary Figure 2

**Additional References**

**Supplementary Methods**

*Laboratory assays*

INSTINCT Nasopharyngeal swabs processing and quantitative real-time PCR:

Throat and nostril were sampled with a single flocked swab which was placed in VTM and transported the same day (except for the day 4 swab which was stored at 2-8°C in the household refrigerator until collection at day 7) by the study team to the Molecular Diagnostic Unit, Imperial College London (MDU). It was stored at 4°C and processed the next working day by extracting Viral RNA using the innuPREP Virus TS RNA 2.0 Kit on a CyBio Felix (Analytik Jena, Jena, Germany), following the manufacturer’s instructions. A multiplex quantitative real-time PCR was used for the E gene and RNase P assay, using the TaqMan Fast Virus 1-Step Master Mix (Thermo Fisher Scientific, Waltham, MA, USA), with the addition of a SARS-COV-2 positive control and negative control. Samples with adequate RNAse P RNA and an E gene Ct <36.5 (which equates to 5 RNA copies per PCR reaction, i.e. 1 E gene RNA copy per μl RNA) were reported as SARS-CoV-2-positive. Participants were deemed PCR-positive if they had any PCR-positive URT-swab in the first 7 days post-enrolment.

ATACCC Nasopharyngeal swabs processing and quantitative real-time PCR:

ATACCC samples were collected by couriers on the same day they were taken and delivered to UK Health Security Agency (UKHSA) Colindale for quantitative real-time PCR testing. Automated extraction of viral RNA from swabs was performed after aliquoting Universal Transport Medium (UTM) (Copan Diagnostics, Murrieta, CA, USA; or MANTACC, Guangdong, China) into lysis buffer and adding internal control. Samples were run on a triplex real-time PCR assay using TaqPath™ 1-Step Multiplex Master Mix (Thermo Fisher Scientific, Waltham, MA, USA) to amplify the targets over 40 cycles, which specifically detects SARS-CoV-2 in the ORF1Ab assay target, and Sarbecoviruses including SARS CoV-2 in the E gene target. SARS-CoV-2 positivity was assigned if either ORF1ab or E gene was detected at Ct<35, or if both targets were detected at Ct between 35 and 40. Participants were deemed PCR-positive if they had any PCR-positive URT-swab in the first 7 days post-enrolment.

Serology:

10mL of serum was collected, centrifuged at 2000g for 10 minutes and divided into 500μl aliquots, which were frozen at -20°C. Antibodies (IgG and IgM) to SARS-CoV-2 receptor binding domain (anti-RBD) was measured using a two-step double antigen binding assay (DABA) (Imperial College London, London, UK) with recombinant S1 antigen on the solid-phase and labelled recombinant RBD as detector in the fluid-phase, as previously described (*1*) (Patent Application No. 2011047.4 for “SARS-CoV-2 antibody detection assay”). Samples with DABA value of 1 or higher were considered positive.

PBMC isolation:

30mL of whole blood was collected in Lithium Heparin tubes. Within 12 hours of receipt, PBMCs were isolated using Ficoll density gradient centrifugation and aliquoted at 10x10^6^ PBMC/ml. Aliquoted PBMCs were then stored at -80°C in Mr. Frosty freezer containers overnight and transferred to liquid nitrogen before use in subsequent assays.

*Further sociodemographic and clinical data*

Vaccination status of participants was obtained by linking case data to the National Immunisation Management System (NIMS) using a unique patient identifier (NHS number) or combinations of NHS number, forename, first initial, surname, date of birth, and postcode. Participants that were vaccinated before or during the study were excluded from the cohort.

Questionnaires assessed the presence/absence of the following, considered as comorbidities:

- Chronic cardiac disease, including congenital heart disease (not hypertension)
- Obesity (as defined by clinical staff)
- Chronic obstructive pulmonary disease (COPD)
- Asthma (physician diagnosed)
- Diabetic
- Chronic kidney disease
- Rheumatologic disorder
- Liver disease
- Malnutrition
- Dementia
- Chronic neurological disorder
- Cancer
- Chronic hematologic disease
- AIDS / HIV

**Supplementary Tables**

**Table S1. Demographic characteristics of the 40 recently-infected contacts** ^1^BMI was not recorded for participants under the age of eighteen. ^2^Three cases of asthma, and one of Hypothyroidism. ^3^ Participants that provided less than 5 consecutive daily symptom diaries had their peak symptom scores excluded.

| **Demographic Characteristics** | | **Total (n=40)** |
| --- | --- | --- |
| **Biological sex** | Female (%) | 17 (42.5) |
|  | Male (%) | 23 (57.5) |
| **Age** | Median (IQR) | 35 (28 - 48) |
|  | < 18 (%) | 2 (5) |
|  | 18 - 49 (%) | 29 (72.5) |
|  | 50 - 64 (%) | 8 (20) |
|  | ≥ 65 (%) | 1 (2.5) |
| **BMI^1^** | Median (IQR) | 23.5 (21.7 - 26.7) |
|  | < 18.5 kg/m^2^ (%) | 0 |
|  | 18.5 to < 25 kg/m^2^ (%) | 22 (55) |
|  | 25 to < 30 kg/m^2^ (%) | 12 (30) |
|  | ≥ 30 kg/m^2^ (%) | 3 (7.5) |
|  | Unknown (%) | 3 (7.5) |
| **Ethnicity** | White (%) | 37 (92.5) |
|  | Non-white (%) | 1 (2.5) |
|  | Unknown (%) | 2 (5) |
| **Presence of co-morbidities^2^** | Yes (%) | 4 (10) |
|  | No (%) | 34 (85) |
|  | Unknown (%) | 2 (5) |
| **Provided symptom diary data^3^** | Yes (%) | 38 (95) |
|  | No (%) | 2 (5) |

**Table S2**. **Daily symptom diary questionnaire.** Contribution of each symptom to the symptom burden score is detailed in the third column, detailing the value of each of them if present, and for some, to what extent. The total burden score is calculated as the summed scores of all present symptoms, divided by the maximum possible score (46.5). The symptom order in the diary is shown in the right-hand column.

| **Type** | **Symptom diary question** | **Answers (points)** |
| --- | --- | --- |
|  | *In the last 24 hours, have you…* |  |
| Canonical definition | felt feverish? | Yes (1.5) / No (0) |
|  | had a persistent cough lasting for more than 4 hours? | Mild (1) / Moderate (2) / Severe (3) / No (0) |
|  | coughed up phlegm or mucous? | Yes (1.5) / No (0) |
|  | had loss or change of smell or taste? | Yes (1.5) / No (0) |
| Lower Respiratory | felt unusually breathless? | Mild (1) / Moderate (2) / Severe (3) / No (0) |
|  | had a wheeze? | Yes (1.5) / No (0) |
|  | coughed blood? | Yes (1.5) / No (0) |
|  | had chest pain? | Mild (1) / Moderate (2) / Severe (3) / No (0) |
| Upper Respiratory | had a runny or blocked nose? | Yes (1.5) / No (0) |
|  | had a sore throat? | Mild (1) / Moderate (2) / Severe (3) / No (0) |
|  | had red or irritated eyes? | Mild (1) / Moderate (2) / Severe (3) / No (0) |
|  | had a hoarse voice? | Yes (1.5) / No (0) |
| Gastrointestinal | had unusual tummy pains? | Yes (1.5) / No (0) |
|  | had loss of appetite? | Mild (1) / Moderate (2) / Severe (3) / No (0) |
|  | felt sick or vomited? | Mild (1) / Moderate (2) / Severe (3) / No (0) |
|  | had diarrhoea? | Mild (1) / Moderate (2) / Severe (3) / No (0) |
| Systemic | had a headache? | Mild (1) / Moderate (2) / Severe (3) / No (0) |
|  | had unusual muscle aches? | Mild (1) / Moderate (2) / Severe (3) / No (0) |
|  | felt tired or generally unwell? | Mild (1) / Moderate (2) / Severe (3) / No (0) |
|  | been confused? | Yes (1.5) / No (0) |

**Table S3. Peptides comprising the cross-reactive pool used for FLISpot stimulation.** Cross-reactive epitopes between SARS-CoV-2 and endemic human coronaviruses were previously identified in the works of Kundu et al. and Nelde et al., using in silico prediction and in vitro expansion, respectively.

| **Sequence** | **Target** | **MHC-restriction** | **HLA-alleles** |
| --- | --- | --- | --- |
| QYIKWPWYIW | Spike | MHC-I | HLA-A*24:02 |
| EAEVQIDRLI | Spike | MHC-I | HLA-B*49:01 |
| KLIANQFNSA | Spike | MHC-I | HLA-A*02:03 |
| RLITGRLQSL | Spike | MHC-I | HLA-A*02:03 |
| RSFIEDLLF | Spike | MHC-I | HLA-B*58:01 |
| SFIEDLLFNKV | Spike | MHC-I | HLA-A*02:01;  HLA-A*02:06 |
| SVLNDILSRL | Spike | MHC-I | HLA-A*02:03 |
| VQIDRLITGR | Spike | MHC-I | HLA-A*68:01 |
| VVNQNAQAL | Spike | MHC-I | HLA-A*26 |
| NQKLIANQFNSAIGK | Spike | MHC-II | HLA-DRB1*13:02 |
| QKFNGLTVLPPLLTD | Spike | MHC-II | HLA-DRB1*01:01 |
| SSNFGAISSVLNDIL | Spike | MHC-II | HLA-DRB1*01:01 |
| KRSFIEDLLFNKVTL | Spike | MHC-II | HLADPA1*01:03; HLA-DPB1*02:01 |
| TTDPSFLGRY | Orf1 | MHC-I | HLA-A*01 |
| KLFAAETLK | Orf1 | MHC-I | HLA-A*03 |
| TPKYKFVRI | Orf1 | MHC-I | HLA-B*08 |
| DLKGKYVQI | Orf1 | MHC-I | HLA-B*08 |
| IEYPIIGDEL | Orf1 | MHC-I | HLA-B*40 |
| LDDFVEIIKSQDLSV | Orf1 | MHC-II | HLA-DRB1*11 |
| WVLNNDYYR | Orf1 | MHC-I | HLA-A*68:01;  HLA-A*31:01;  HLA-A*33:01 |
| YRLANECAQV | Orf1 | MHC-I | HLA-A*02:03;  HLA-A*02:01;  HLA-A*02:06 |
| FVDGVPFVV | Orf1 | MHC-I | HLA-A*02:06;  HLA-A*02:01 |
| HEFCSQHTM | Orf1 | MHC-I | HLA-B*40:01 |
| FVSLAIDAY | Orf1 | MHC-I | HLA-B*35:01 |
| VLYYQNNVF | Orf1 | MHC-I | HLA-B*15:01 |
| SVFNICQAV | Orf1 | MHC-I | HLA-A*68:02;  HLA-A*02:06;  HLA-A*02:03 |
| RILGAGCFV | Orf1 | MHC-I | HLA-A*02:06 |
| TQMNLKYAI | Orf1 | MHC-I | HLA-A*02:06 |
| NVNRFNVAI | Orf1 | MHC-I | HLA-A*68:02 |
| SLAIDAYPL | Orf1 | MHC-I | HLA-A*02:01 |
| AAVDALCEK | Orf1 | MHC-I | HLA-A*11:01 |
| KDGIIWVATEGALNT | Nucleocapsid | MHC-II | HLA-DRB1*01, DRB1*04,  DRB1*11 |
| GTWLTYTGAIKLDDK | Nucleocapsid | MHC-II | HLA-DRB1*01,  DRB1*07,  DRB1*15 |
| RWYFYYLGTGPEAGL | Nucleocapsid | MHC-II | HLA-DRB1*04,  HLA-DRB1*01:01 |
| ASWFTALTQHGKEDL | Nucleocapsid | MHC-II | HLA-DRB1*04, DRB1*11 |
| ASAFFGMSRIGMEVT | Nucleocapsid | MHC-II | HLA-DRB1*01,  DRB1*04, DRB1*07,  DRB1*11 |
| LLLLDRLNQLESKMS | Nucleocapsid | MHC-II | HLA-DRB1*04, DRB1*15 |
| KPRQKRTA | Nucleocapsid | MHC-I | HLA-B*08 |
| PRWYFYYLGT | Nucleocapsid | MHC-I | HLA-B*08:01; HLA-B*14:02 |
| RTFKVSIWNLDY | ORF6 | MHC-I | HLA-A*01:01 |
| YEGNSPFHPL | ORF7, ORF4 | MHC-I | HLA-B*40 |
| FYVYSRVKNLNSSRV | Envelope | MHC-II | HLA-DRB1*04, DRB1*11 |
| IWNLDYIINLIIKNL | ORF6 | MHC-II | HLA-DRB1*04,  DRB1*07,  DRB1**15 |
| QEEVQELYSPIFLIV | ORF7 | MHC-II | HLA-DRB1*01, DRB1*07 |
| SKWYIRVGARKSAPL | ORF8 | MHC-II | HLA-DRB1*01, DRB1*11 |

**Supplementary Figure 1**

**
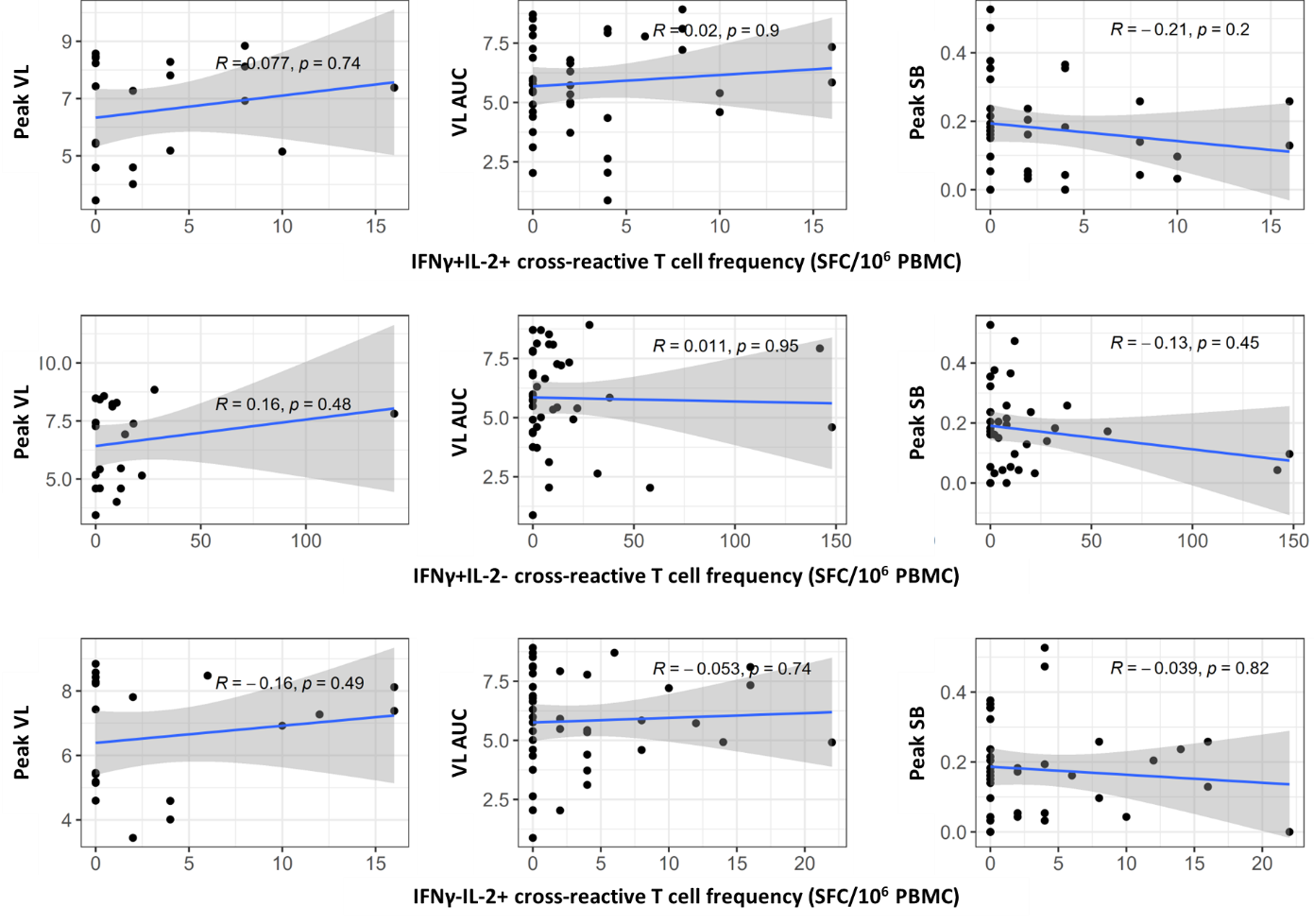
**

**Supplementary Figure 1. Pre-existing cross-reactive T cells do not correlate significantly with COVID-19 outcome**

A. Spearman’s correlation analysis of associations between fequencies of cross-reactive peptide-responsive T cells at d0 and peak viral load (n=21). B. Spearman’s correlation analysis of associations between fequencies of cross-reactive peptide-responsive T cells at d0 and peak symptom burden (n=38).

**Supplementary Figure 2**

**
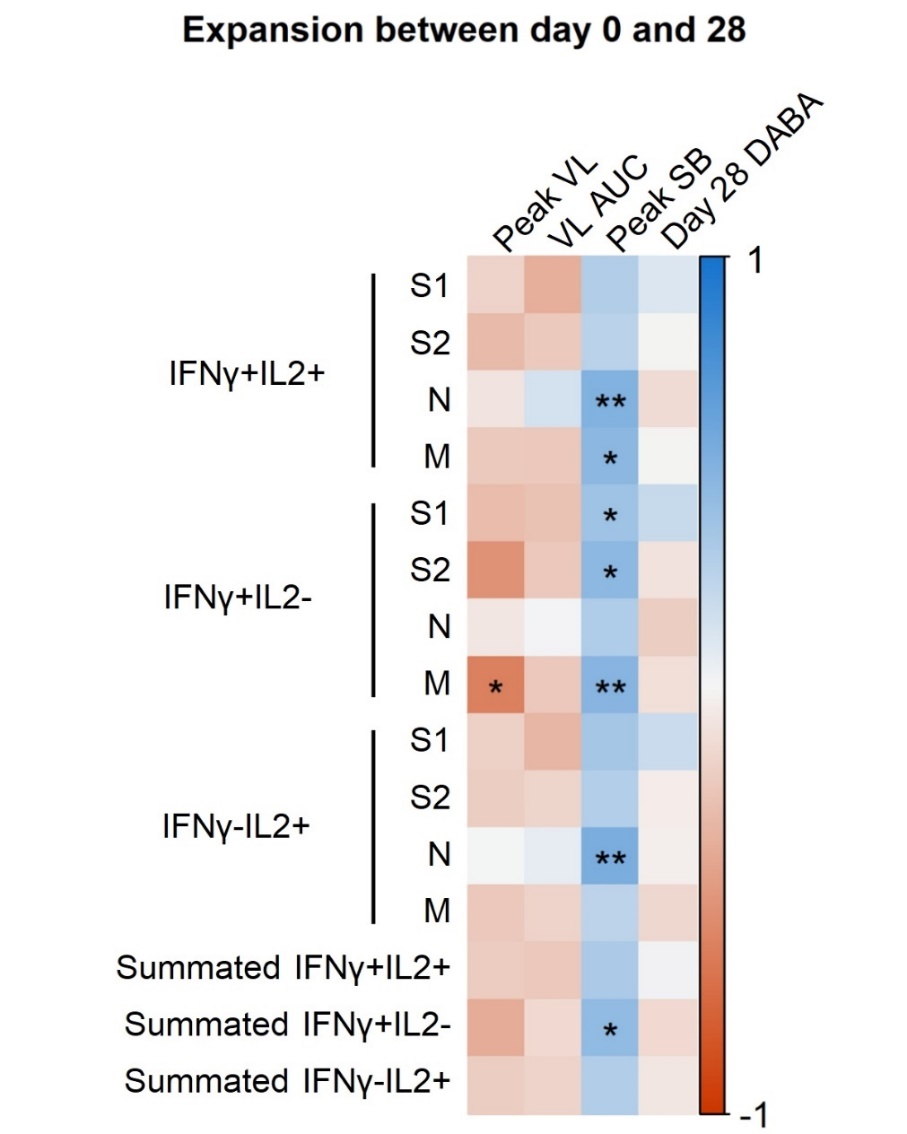
**

**Supplementary Figure 2. Total expansion of *de novo* antigen-specific T cells positively associates with higher symptom burden.** Spearman’s correlations between the frequencies of T cells expanded from d0 to 28 and infection outcomes are shown. Summated denotes the sum of responses to the S, M and N pools by the respective subset.

Statistically significant correlations are indicated by asterisks (* p ≤ 0.05, ** p ≤ 0.01, *** p ≤ 0.001, **** p ≤ 0.0001.)

1. C. Rosadas *et al.*, Detection and quantification of antibody to SARS CoV 2 receptor binding domain provides enhanced sensitivity, specificity and utility. *J Virol Methods* **302**, 114475 (2022).
